# Impact of moderate environmental heat stress during running exercise on circulating markers of gastrointestinal integrity in endurance athletes

**DOI:** 10.14814/phy2.70305

**Published:** 2025-04-02

**Authors:** Thomas Beiter, Gunnar Erz, Anna Würden, Andreas M. Nieß

**Affiliations:** ^1^ Department of Sports Medicine University Hospital Tübingen Tübingen Germany

**Keywords:** body temperature, exercise, gastrointestinal integrity, heat stress, intestinal permeability, leaky gut

## Abstract

In the present study, we aimed to determine the effect of moderate ambient heat stress on exercise‐provoked patterns of “leaky gut” biomarkers and stress markers in well‐trained athletes. Eleven triathletes performed a strenuous 1‐h treadmill run, both under normal ambient conditions (N, 18–21°C) as well as under moderate heat environmental conditions (H, 28–30°C). Core body temperature (Tc), heart rate (HR), and rating of perceived exertion (RPE) significantly increased under both conditions, with significantly higher values during and after the H run. We observed a significant main effect of acute exercise on circulating leukocyte numbers, release of cell‐free human DNA (cfDNA) but not bacterial DNA (bacDNA), and on plasma levels of intestinal fatty‐acid binding protein (I‐FABP), lipopolysaccharide‐binding protein (LBP), endotoxin (LPS), and D‐lactate. Exercising under H conditions accelerated the mobilization of circulating neutrophils and lymphocytes, and significantly affected the release of cfDNA, D‐lactate, I‐FABP, creatinine, and blood potassium levels. Multiple correlation analysis revealed a significant association between Tc, max and exercise‐provoked release of cfDNA (*r* = 0.583, *p* = 0.012) as well as with I‐FABP (*r* = 0.554, *p* = 0.026). Our data indicate that acute exercising and heat stress may not only affect paracellular but also transcellular intestinal permeability.

## 1 INTRODUCTION

Heatwaves of increasing severity and duration are the greatest, direct, climate‐related public health threat of the 21st Century (Romanello et al., 2022). Vulnerable groups most at risk include the very old, the very young, those with existing health problems or disabilities, and especially those at the bottom of the socioeconomic ladder. But vulnerability further extends to a broad range of apparently fit and healthy people that either work outside or perform strenuous outdoor physical activity (Morris et al., 2021). This necessarily also includes both professional and recreational sports. As many sport events are held during the hottest months of the year, heat exhaustion, heat stroke, and heat‐related illnesses have by now become major health issues affecting athletes from various disciplines (Racinais et al., 2023).

When exercising in the heat, aerobic performance is reduced and the cardiovascular system is put under considerable strain (Periard et al., 2021). How well the body is able to cope with increased ambient temperatures depends on numerous factors, such as body composition, cardiorespiratory fitness, blood volume, electrolyte homeostasis, skin circulation, or the ability to secrete sweat (Cramer et al., 2022). Even though endurance‐trained persons naturally have a better heat tolerance compared to the general population and also to other sport disciplines, thermoregulatory responses during exercise in the heat are nonetheless individually determined and the thermoregulatory capacity may vary considerably among these athletes (Corbett et al., 2018; De Korte et al., 2021; Gibson et al., 2020; Heled et al., 2004; Racinais et al., 2022). In recent years, a growing body of research has revealed evidence that the individual tolerance to extreme temperatures might be closely associated with the intestine and its microbiota (Cinca‐Morros & Alvarez‐Herms, 2024).

Thermal stress induces a robust hyperadrenergic state with prolonged sympathetic activation that reduces overall gastrointestinal functional capacity through suppressing myenteric and submucosal plexus activity (De Oliveira et al., 2014). In an effort to dissipate heat from the body's core to the skin surface, activation of sympathetic cholinergic vasodilator nerves triggers peripheral vasodilation and sweating. The resulting sizeable increase in peripheral circulation reduces blood flow to the internal organs. This phenomenon becomes amplified in the exercising body as the accelerated demands of the working muscle also require additional supporting blood supply. As a consequence, blood flow becomes rapidly redistributed away from the less active gastrointestinal system, resulting in reduced total splanchnic perfusion (Van Wijck et al., 2011). Local gastrointestinal ischemia and hypoxia in conjunction with increased heat load then provoke activation of heat shock and oxidative stress responses in the intestinal epithelium. Immediate implications are changes in tight junction architecture, disturbed intestinal permeability, and nutrient malabsorption. When thermal and oxidative stress to the enteric tissue sustain or accelerate, intestinal integrity may become compromised, and the balance between the gut microbiome and the immune system will become challenged (Ribeiro et al., 2021). Disturbance of intestinal cell function and cohesion leading to impaired barrier function is commonly referred to as “leaky gut” (Dmytriv et al., 2024). As a consequence, potentially harmful luminal contents, like bacterial antigens, bacterial decomposition products, or toxic substances could cross the gut mucosal barrier. Activation of the mucosal immune system would then provoke acute abdominal pain and diarrhea. Should perturbations to gastrointestinal integrity or function exaggerate or persist, severe acute or chronic health complications may arise with short‐ and long‐term implications for athletic performance (Costa et al., 2020; Ogden, Child, et al., 2020).

Gastrointestinal complaints during and after endurance events are common problems faced by many athletes (De Oliveira et al., 2014). Several studies have demonstrated elevated levels of “leaky gut” biomarkers after prolonged exercise performed either in laboratory settings or in outdoor competitions (Ogden, Child, et al., 2020). The range of blood markers commonly used to assess barrier integrity include intracellular constituents of enterocytes like intestinal fatty‐acid binding protein (I‐FABP), hepatic acute phase proteins like the lipopolysaccharide (LPS)‐binding protein (LBP), or microbial‐derived products and metabolites like bacterial endotoxin (LPS), bacterial DNA (bacDNA), or bacterial D‐Lactate. Albeit increased core body temperature (Tc) appears to be a determinant factor underlying the emergence of exercise‐provoked gastrointestinal symptoms (Henningsen, Mika, et al., 2024), reported correlations between blood‐borne markers of intestinal distress, intestinal permeability and observed symptoms emerge to be generally weak and inconsistent (Jeukendrup et al., 2000; Karhu et al., 2017; Pugh et al., 2017; Roca Rubio et al., 2024; Van Nieuwenhoven et al., 2004). As the term “leaky gut” is no registered trademark, it is often used to describe apparently connected but not necessarily causally related, coexisting, or interchangeable phenomena like “increased intestinal permeability”, “disturbed barrier function”, “perturbed gut integrity”, or “enhanced microbial translocation”. Thus, the connection between exercise‐induced hyperthermia, “leaky gut syndrome” and the etiology and pathophysiology of gastrointestinal dysfunction in endurance sports clearly warrants further investigations.

In the present study, we determined exercise‐provoked patterns of physiological and humoral markers commonly associated with heat stress and gastrointestinal distress in well‐trained triathletes following an individually adjusted 1‐h treadmill run, both under normal ambient condition (N, 18–21°C) as well as under moderate heat environmental conditions (H, 28–30°C). We sought to assess which parameters are released or do increase as part of the normal physiological and immunological response to an acute bout of vigorous endurance exercise, which parameters become affected when ambient temperature is elevated, and which parameters show correlative connections with the increasing Tc.

## 2 MATERIALS AND METHODS

### 2.1 Participants and general procedures

Eighteen triathletes (16 males, 2 females) with an active competition history volunteered to participate in this study. All of them were familiar with laboratory stress testing and treadmill running. Exclusion criteria were as follows: nicotine consumption, current or previous alcohol or drug addiction, acute medication, inflammatory or infectious diseases, intestinal diseases, previous surgical interventions on the stomach or intestines, chronic diseases, and restrictions on fitness for competition.

The study was conducted in accordance with the Declaration of Helsinki and approved by the local ethics committees of the University of Tübingen (approval number: 008/2022BO2). All participants provided signed, written informed consent.

To characterize subjects, height and weight were measured using standard techniques, and body composition was assessed using a bioelectrical impedance analysis system (InBody 4.0, InBody Europe B.V., Eschborn, Germany). For the determination of maximal oxygen uptake (VO_2_max) and lactate thresholds (LTs), participants undertook a graded exercise test (start, 6 km/h; increment, 2 km/h; duration, 3 min) to volitional exhaustion on a motorized treadmill (Saturn, h/p/cosmos, Nussdorf‐Traunstein, Germany). Capillary blood lactate concentrations from the earlobe were measured before the test as well as during the last 20 s of each stage and right after volitional exhaustion (Biosen S‐Line, EKF diagnostics, Barleben, Germany). The LT was determined as the minimal lactate equivalent (lactate/performance quotient), and the individual anaerobic threshold (IAT) was calculated 1.5 mmol/L above the lactate concentration of the LT according to Dickhuth et al. (1999). Heart rate (HR) and electrocardiogram were constantly recorded (12‐channel PC ECG, Custo med GmbH, Ottobrunn, Germany). Blood pressure was recorded before and immediately after volitional exhaustion. Breath‐by‐breath pulmonary gas exchange and ventilation were measured using a metabolic cart (MetaLyzer, CORTEX Biophysics GmbH, Leipzig, Germany). The VO_2_max was determined using the plateau phase “leveling off” of the O_2_ kinetics as the main criterion.

### 2.2 Laboratory exercise test

**FIGURE 1:**
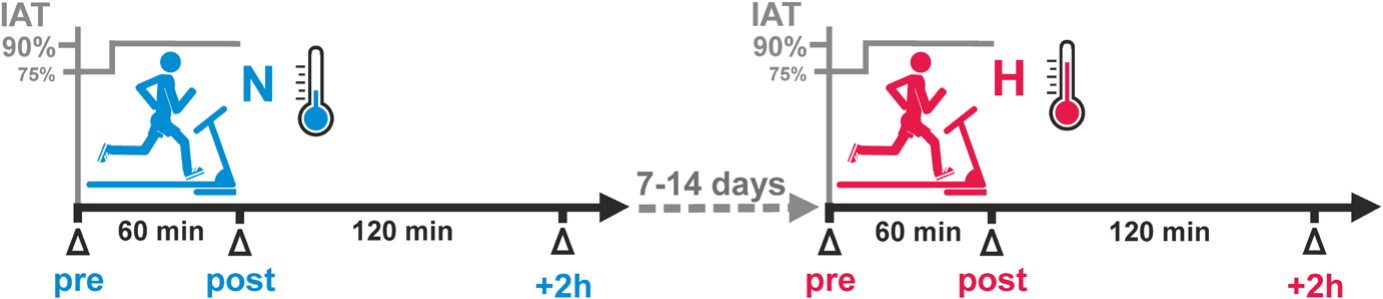
Schematic illustration of the paired study setup. Eleven triathletes (10 males, 1 female) performed two 1‐h treadmill exercise sessions that were adjusted to the individual anaerobic threshold (IAT); one under temperate ambient condition (N, 19.2 ± 0.9°C) and one under moderate heat condition (H, 28.7 ± 0.7°C). Exercise started with a 10‐min warm‐up followed by 50 min of main exercise at a speed required to run at 90% of the IAT. Blood samples were collected immediately before (pre), immediately after (post), as well as at 2 h (+2 h) after the exercise tests.

Within 4 weeks from the initial physiological diagnostic, the study participants performed a first treadmill session in an air‐conditioned laboratory. Participants were instructed to refrain from physical activity for 24 h and food intake for 3 h prior to the exercise test. An ingestible telemetric pill (e‐Celsius, BodyCAP, Hérouville Saint‐Clair, France) was provided to each athlete the day before the test, and participants were instructed to swallow the pill at 1 h before going to sleep. On the day of the test, the participants entered into the climatic laboratory that was already at the programmed temperature. Participants immediately started running without initial resting acclimation time. The exercise test consisted of a 1‐h running bout on a motorized treadmill (Saturn, h/p/cosmos). Exercise started with 10 min of warm‐up at an initial speed required to run at 75% of the IAT, followed by 50 min of main exercise at a constant speed corresponding to 90% of IAT. The first exercise test was performed at normal ambient temperature (18–21°C, N). A second exercise session under moderate environmental heat stress (H) followed after 7–14 days at an ambient temperature set to 28–30°C (Figure 1).

During the runs, participants were allowed to drink ad libitum from a weighed water bottle (prewarmed to body temperature). Immediately before and after the exercise bout, participants were weighed in underwear, and total body sweat loss was calculated from the differences in body weight corrected for fluid intake.

Tc data from the telemetric ingestible pill were transmitted every 30 s and displayed in real time on an external receiver. Every 5 min during the test, HR was recorded by use of a polar heart watch system (Polar Electro GmbH, Büttelborn, Germany) and participants were asked to rate their level of exertion using Borg's Rating of Perceived Exertion (RPE) scale (Borg, 1970). Blood lactate values were obtained from the earlobe immediately before and after the exercise test.

Baseline blood samples from the antecubital vein were obtained immediately before the exercise test (pre). Post‐exercise samples were collected immediately after (post) as well as 2 h (+2 h) after the exercise session. Serum and plasma samples for experimental analysis were instantly obtained by centrifugation (1800 g, 10 min, 4°C), snap frozen in liquid nitrogen, and stored at −80°C until further processing. During the 2‐h recovery period, participants were allowed to continue normal day activities but were restricted from any strenuous activities or exercise.

From the 18 included probands, a total of 16 subjects (14 males, 2 female) absolved the complete exercise test under H condition. Two subjects prematurely terminated the protocol due to unspecified fatigue and, therefore, were excluded from the analysis. Due to a limitation of the air‐conditioning system during the exercise test of five participants, ambient temperature in the N condition trial significantly raised above the 21°C limit. Therefore, only data from 11 participants (10 males, 1 female) were amenable for paired analysis to compare differential responsiveness between N and H conditions. The anthropometric data and performance characteristics of the included study participants are given in Table 1.

**TABLE 1:** Physiological characteristics and anthropometric data of study participants.

Variables		
Gender (male/female)	10/1	(14/2)
Age (years)	33.6 ± 6.7	(32.9 ± 6.1)
Height (cm)	180.0 ± 6.0	(180.1 ± 5.6)
Body weight, BW (kg)	71.4 ± 9.3	(71.5 ± 8.3)
Body mass index, BMI (kg/m^2^)	22.0 ± 1.9	(22.0 ± 1.8)
Body fat (%)	9.8 ± 3.7	(11.3 ± 4.7)
Muscle mass (kg)	38.3 ± 4.7	(37.1 ± 5.4)
VO_2_max (mL/min/kg)	59.5 ± 6.7	(58.5 ± 7.3)
Speed at IAT (km/h)	14.7 ± 1.5	(14.3 ± 1.5)

*Note*: Main values represent data of 11 subjects that completed the exercise test both under standardized normal ambient temperature (N) as well as under moderate heat stress (H); values in brackets represent the total number of subjects (*n* = 16) that completed the exercise test under H condition. Values are given as mean ± SD.

### 2.3 Determination of clinical and laboratory parameters

Blood cell counts as well as routine laboratory parameters (aspartate aminotransferase, AST; alanine aminotransferase, ALT; serum creatinine, SCr; creatine kinase, CK; C‐reactive protein, CRP; electrolytes; gamma‐glutamyl transferase, GGT; lactate dehydrogenase, LDH; urea) were measured under accredited conditions at the central laboratory of the University Hospital of Tübingen.

### 2.4 Biomarker analysis

#### 2.4.1 Fatty acid‐binding protein (I‐FABP) and LPS‐binding protein (LBP)

Plasma concentrations of I‐FABP and LBP were measured by commercially available enzyme‐linked immunosorbent assay kits (Hycult Biotech, Beutelsbach, Germany; catalogue #HK406 and #HK315) on freshly thawed plasma. For the I‐FABP assay, heparin plasma samples were diluted 1/10 in reagent diluent, while for the LBP assay, EDTA plasma samples were diluted 1/1500. All samples were run in duplicates according to the manufacturer's instructions. Samples from the same individual were performed simultaneously on one plate. Optical density was determined using an Infinite 200 PRO plate reader (Tecan Group Ltd., Männedorf, Switzerland) set to 450 nm with a correction filter at 570 nm.

#### 2.4.2 Bacterial D‐lactate

The content of D‐lactate in serum was determined using Cayman Chemicals' fluorescence‐based D‐lactate detection kit (Biomol GmbH, Hamburg, Germany; catalogue #Cay700520), as per manufacturer's instructions. Briefly, the concentration of D‐lactate from 500 μL deproteinated serum sample supernatant was calculated by monitoring NADH derived from the enzymatic conversion of D‐lactate to pyruvate in the presence of a fluorometric substrate, with an excitation wavelength of 535 nm and emission at 590 nm.

#### 2.4.3 Limulus amebocyte lysate (LAL) assay

Plasma endotoxemia (endotoxin unit per milliliter, EU/mL) was determined by using the Lonza Kinetic‐QCL assay (Fisher Scientific GmbH, Schwerte, Germany; catalogue #50‐650U). To avoid contamination, all procedures used pyrogen‐free materials, including pyrogen‐free tips. Sample preparation was performed as described by Laugerette et al. (2015). Briefly, heparin plasma samples were diluted 1:10 in endotoxin‐free water and heat treated at 70°C for 10 min to inactivate endotoxin‐neutralizing agents. Then samples were subjected to an ultrasonic bath (37°C, 10 min) and vortexed for 1 min to enhance recovery of measurable LPS activity. Calibrators and reagents were prepared according to the instructions provided by the manufacturer. Plate reading was performed using the Infinite 200 PRO plate reader. Prior to the addition of reagent, samples and standards were pre‐incubated for 10 min at 37°C. Kinetics were then monitored at 405 nm every 2 min and 30 s for 2 h in a constant temperature of 37°C. The increase in signal of calibrators and samples was plotted against time; both delta signal and concentration were log transformed. The slopes of the calibrator curves were modeled with a 4PL equation, and the concentrations of the samples were interpolated on this curve according to the operation instructions.

#### 2.4.4 Circulating DNA fragments

Circulating DNA fragments were extracted from 500 μL of EDTA plasma using the Norgen Cell‐Free Circulating DNA Purification Mini Kit (BioCat GmbH, Heidelberg, Germany; catalogue #Dx55100‐NB) according to the manufacturer's protocol, with a final elution volume of 50 μL. Human circulating cell‐free (cfDNA) equivalents were quantified by SYBR green real‐time PCR that targeted an 88‐bp fragment of the chromosomal myostatin (MSTN) gene locus (primer sequences: 5′‐TTG GCT CAA ACA ACC TGA ATC C‐3′ and 5′‐TCC TGG GAA GGT TAC AGC AAG‐3′), as described previously (Beiter et al., 2011). Briefly, sample eluates (2.5 μL) were run in duplicates on a CFX‐96 thermocycler (BioRad, Munich, Germany) using SsoFast EvaGreen Supermix (BioRad; catalogue #1725202) in a total reaction volume of 15 μL. PCR was performed for 40 cycles at 98°C for 5 s and at 59°C for 15 s after initial incubation at 98°C for 30 s. Product specificity was confirmed by melting curve analysis. DNA levels were calculated from a standard curve generated from serial dilutions of a genomic calibrator by plotting the threshold cycle value against the logarithm of calibrator copy numbers. Total quantities of cfDNA are given as genome equivalents (GE) per mL of plasma, with one GE being defined as the amount of the target sequence contained in a single haploid cell.

For quantification of circulating bacterial DNA (bacDNA), extracted DNA samples were analyzed by use of the Femto™ Bacterial DNA Quantification Kit (Zymo Research Europe GmbH, Freiburg, Germany; catalogue #E2006) in a total volume of 20 μL, including 2.5 μL sample and qPCR premix with a universal primer pair targeting conserved stretches flanking the hypervariable region of the bacterial 16S rRNA gene. The thermal profile used for the reaction included denaturation at 95°C for 10 min, followed by 40 cycles of denaturation at 95°C for 30 s, annealing at 50°C for 30 s, and extension at 72°C for 1 min. Standard curves were obtained using a 10‐fold dilution series of bacterial DNA standards ranging from 20 ng to 20 fg per reaction. Preparation of standard curves, sample template addition, and PCR runs were performed in separate working areas using dedicated pipettes and filter‐sealed tips. Negative controls, in which ultrapure water was added instead of DNA eluate, were run in each plate. BacDNA levels are given as pg per mL plasma.

### 2.5 Statistical analysis

All statistical analyses were performed using JMP software (JMP version 16.0.0, JMP Statistical Discovery LLC, Cary, NC, USA). Comparisons were made after assessing normal distribution using Shapiro–Wilk test and visual inspection of Q–Q plots. Skewed data were log‐transformed before use in subsequent analyses. Homogeneity of variance assumption was checked by Levene's test. To examine the influence of ambient temperature on exercise‐provoked changes in blood markers, a two‐way repeated‐measures analysis of variance (ANOVA) was used with condition (H, N) and time point (pre, post, +2 h) as within‐subject factors. The magnitude of the differences was assessed with partial eta squared values ($\eta_{p}^{2}$). Where significant main or interaction effects were identified, post hoc Holm‐Bonferroni step‐wise corrected paired *t*‐tests were used to determine the location of variance. For variables that did not meet the equality of variance assumption, Friedman's test and post hoc Wilcoxon corrections were used instead. Relationships between variables were assessed by use of Pearson's correlation coefficient. Statistical significance was accepted at the alpha level of *p* ≤ 0.05. Data are reported as mean ± SD in case of normal distribution or as median [interquartile range] in case of non‐normal distribution and for ordinal variables. Mean or median differences are reported with a 95% confidence interval (CI) or first and third quartile [interquartile range], respectively.

## 3 RESULTS

### 3.1 Core temperature, heart rate, perceived exertion, and sweat loss

**FIGURE 2:**
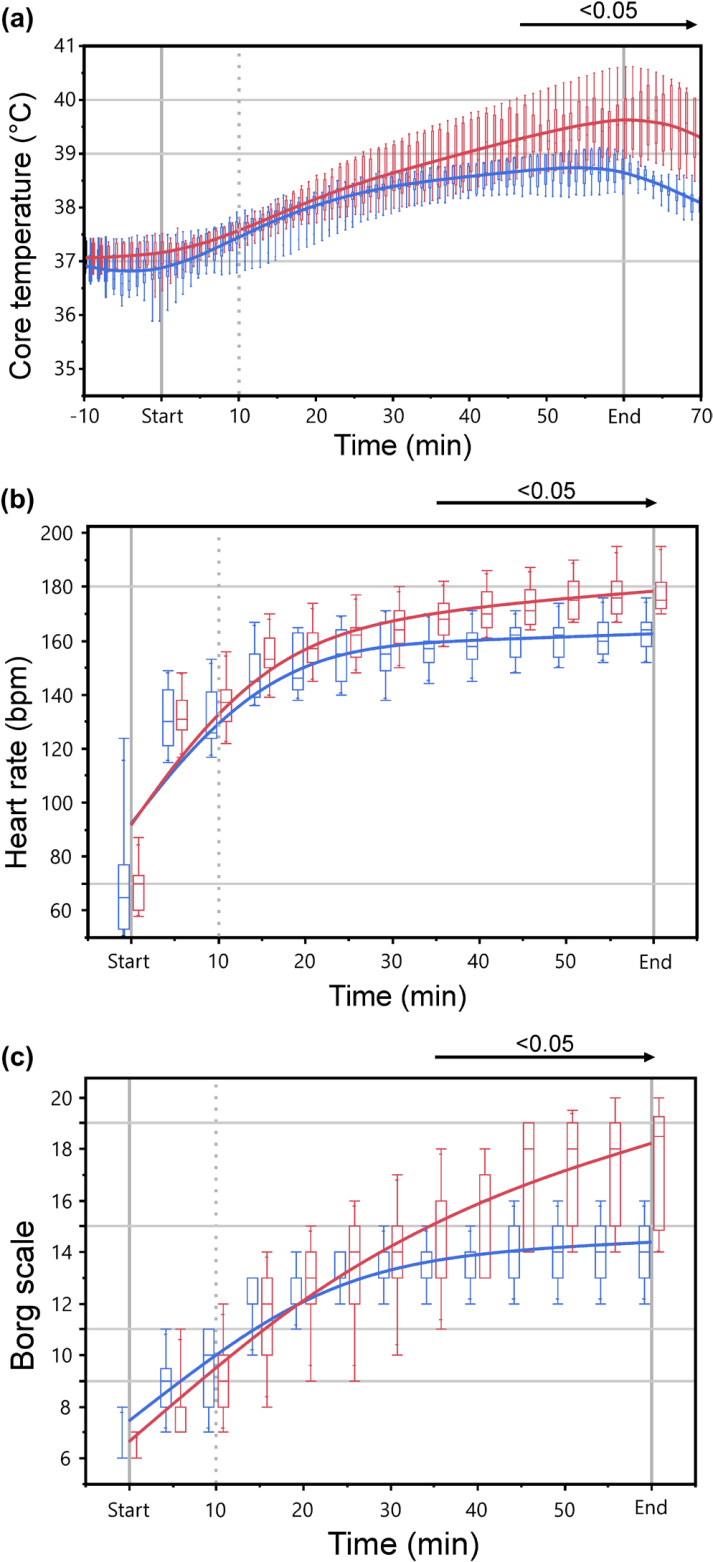
Evolution of core body temperature (Tc, a), heart rate (HR, b), and ratings of perceived exertion (RPE, c) during the 1‐h treadmill session under normal ambient temperature (N, blue) and under moderate heat condition (H, red). Box‐plots represent data points of 11 study participants. The time period with significant pairwise differences between conditions (*p* < 0.05) is indicated by a horizontal arrow.

To assess the within‐subject influence of ambient temperature on exercise‐induced changes in Tc, HR, and RPE, 11 well‐trained triathletes completed the 1‐h treadmill protocol (main exercise speed: 13.3 ± 1.3 km/h), both under temperate ambient conditions (N, 19.2 ± 0.9°C; relative humidity, 69.2 ± 6.5%) and under moderate environmental heat stress (H, 28.7 ± 0.7°C; relative humidity, 53.7 ± 6.8%). Ambient temperature significantly affected exercise‐induced responses in Tc (time × condition: *F* (12, 120) = 8.79, *p* < 0.001, $\eta_{p}^{2}$ = 0.465), HR (time × condition: *F* (12, 120) = 4.22, *p* < 0.001, $\eta_{p}^{2}$ = 0.317), and RPE (time × condition: *F* (12, 120) = 19.80, *p* < 0.001, $\eta_{p}^{2}$ = 0.645). After 35 min of running, ambient temperature started to significantly impact exercise‐provoked levels of HR and RPE, while significant differences between Tc values became apparent from minute 45 onwards (Figure 2). Maximum Tc (Tc, max) at the end of the run was by 0.9°C higher (CI: 0.6–1.3°C, *p* < 0.001; N, 38.7 ± 0.3°C; H, 39.6 ± 0.5°C) when exercise was performed under H condition. Likewise, maximum HR became increased by 16 bpm (CI: 12–19 bpm, *p* < 0.001; N, 163 ± 7 bpm; H, 179 ± 7 bpm), and maximum RPE became elevated by 4 units ([3, 5], *p* < 0.002; N, 14 [13, 15]; H, 19 [15, 20]). Ambient heat stress during exercise provoked an additional sweat loss of 0.51 kg (CI: −0.17 to −0.84 kg, *p* = 0.007; N, −1.42 ± 0.32; H, −1.93 ± 0.54 kg) compared to exercise at normal temperature.

### 3.2 Impact of ambient temperature on exercise‐provoked changes in hematological and biochemical blood parameters

Blood samples were analyzed for known exercise‐responsive blood components and biochemical markers connected to heat adaptation and gastrointestinal constraints. An overview of observed main and interaction effects provoked by acute exercise and ambient temperature on these markers is given in Table 2. For all analyzed blood samples, levels of CRP remained under the quantification limit (≤0.05 mg/dL) of the routine laboratory test.

**TABLE 2:** Effects of acute exercise and ambient temperature on measured blood variables.

Blood variable	Ambient condition	Exercise			Main effect: Exercise			Main effect: Condition			Interaction		
		Pre	Post	+2 h	*F*	*p*	$\eta_{p}^{2}$	*F*	*p*	$\eta_{p}^{2}$	*F*	*p*	$\eta_{p}^{2}$
Neutrophils (Tsd/μL)	N	2.25 ± 0.8	**3.02 ± 0.9** ^a^	**5.23 ± 1.9** ^a^	**20.7**	**<0.01**	**0.58**	**13.5**	**0.01**	**0.31**	**3.41**	**0.05**	**0.19**
	H	2.46 ± 0.6	**3.44 ± 1.3** ^a^	**6.98 ± 2.0** ^a^, ^b^									
Lymphocytes (Tsd/μL)	N	1.64 ± 0.2	**1.97 ± 0.5** ^a^	1.45 ± 0.3	**15.4**	**<0.01**	**0.51**	**6.50**	**0.02**	**0.18**	**5.90**	**0.01**	**0.28**
	H	1.79 ± 0.5	**2.43 ± 0.4** ^a^, ^b^	**1.36 ± 0.4** ^a^									
Monocytes (Tsd/μL)	N	0.41 ± 0.1	0.38 ± 0.1	0.43 ± 0.1	2.22	0.12	0.13	**6.27**	**0.02**	**0.17**	1.86	0.17	
	H	0.42 ± 0.2	0.41 ± 0.1	0.55 ± 0.1									
cfDNA (GE/mL)	N	159 [101, 198]	**2046 [1336, 3196]** ^a^	108 [75, 139]	**214**	**<0.01**	**0.96**	2.90	0.12	0.22	**3.62**	**0.04**	**0.27**
	H	204 [94, 294]	**3305 [2306, 4895]** ^a^, ^b^	89 [37, 183]									
D‐lactate (μmol/L)	N	76.2 ± 3.6	77.1 ± 5.9	72.9 ± 4.5	**5.40**	**0.01**	**0.26**	**8.00**	**0.01**	**0.21**	**4.94**	**0.01**	**0.25**
	H	75.1 ± 4.9	**83.2 ± 7.5** ^a^, ^b^	75.4 ± 4.8									
I‐FABP (pg/mL)	N	297 [230, 362]	**1102 [800, 2492]** ^a^	**624 [444, 1122]** ^a^	**20.2**	**<0.01**	**0.57**	1.19	0.28	0.04	**3.82**	**0.03**	**0.20**
	H	245 [161, 308]	**1982 [1071, 3135]** ^a^	**985 [769, 1286]** ^a^									
LBP (μg/mL)	N	8.67 ± 2.8	**10.5 ± 2.3** ^a^	9.53 ± 2.5	**30.1**	**<0.01**	**0.75**	2.20	0.17	0.18	4.14	0.10	0.29
	H	6.84 ± 1.5	**11.6 ± 3.4** ^a^	7.38 ± 1.6									
LPS (EU/mL)	N	0.01 [0.01, 0.01]	**0.12 [0.02, 0.26]** ^a^	0.01 [0.01, 0.01]	**213**	**<0.01**	**0.87**	0.86	0.38	0.15	2.91	0.09	0.11
	H	0.01 [0.01, 0.01]	**0.15 [0.02, 0.32]** ^a^	0.01 [0.01, 0.01]									
bacDNA (ng/mL)	N	1.37 [1.06, 1.96]	1.32 [0.92, 3.5]	1.48 [0.97, 1.87]	4.12	0.13	0.11	3.77	0.19	0.16	0.33	0.72	0.02
	H	0.97 [0.79, 1.45]	1.17 [0.78, 1.31]	0.87 [0.74, 1.22]									
Sodium (mmol/L)	N	140 ± 1.8	141 ± 1.6	140 ± 1.1	2.24	0.13	0.18	0.06	0.81	0.01	1.04	0.37	0.09
	H	140 ± 2.5	141 ± 2.8	140 ± 2.1									
Potassium (mmol/L)	N	3.98 ± 0.3	3.99 ± 0.2	4.14 ± 0.3	**4.80**	**0.02**	**0.24**	1.42	0.24	0.04	**6.74**	**0.01**	**0.31**
	H	3.68 ± 0.3	**4.19 ± 0.2** ^a^, ^b^	4.01 ± 0.4									
Creatinine (mg/dL)	N	0.77 ± 0.1	**0.90 ± 0.1** ^a^	**0.87 ± 0.1** ^a^	**44.3**	**<0.01**	**0.81**	**6.11**	**0.03**	**0.38**	**5.73**	**0.01**	**0.37**
	H	0.82 ± 0.1	**1.04 ± 0.1** ^a^, ^b^	**0.97 ± 0.1** ^a^									
LDH (U/L)	N	203 ± 40	**236 ± 41** ^a^	**226 ± 39** ^a^	**79.7**	**<0.01**	**0.89**	0.83	0.39	0.08	0.22	0.81	0.02
	H	210 ± 44	**248 ± 45** ^a^	233 ± 43									
CK (U/L)	N	234 ± 91	**281 ± 94** ^a^	**264 ± 84** ^a^	**52.4**	**<0.01**	**0.84**	0.06	0.97	0.01	1.66	0.21	0.14
	H	226 ± 125	**278 ± 150** ^a^	**272 ± 157** ^a^									
Urea (mg/dL)	N	35.5 ± 7.8	**37.9 ± 6.6** ^a^	**39.9 ± 8.1** ^a^	**25.6**	**<0.01**	**0.72**	0.09	0.77	0.01	0.36	0.70	0.03
	H	35.8 ± 8.3	**38.6 ± 9.2** ^a^	**40.9 ± 9.6** ^a^									
Gamma‐GT (U/L)	N	17.5 ± 4.2	18.7 ± 4.6	19.4 ± 3.9	**9.25**	**0.01**	**0.48**	2.72	0.13	0.21	0.42	0.67	0.04
	H	18.6 ± 5.2	20.3 ± 5.3	19.5 ± 4.5									
AST (U/L)	N	24.8 ± 7.3	**28.6 ± 6.8** ^a^	24.9 ± 6.7	**16.2**	**<0.01**	**0.62**	0.04	0.84	0.01	3.08	0.07	0.24
	H	23.6 ± 6.0	**29.2 ± 5.5** ^a^	27.5 ± 5.6									
ALT (U/L)	N	25.5 ± 5.9	25.2 ± 4.4	23.9 ± 5.4	1.62	0.22	0.14	0.09	0.77	0.01	2.12	0.15	0.16
	H	24.2 ± 6.7	26.7 ± 6.6	25.1 ± 6.8									

*Note*: Measured values represent mean ± SD in the case of normal distribution or medians [interquartile range] in the case of non‐normal distribution, respectively, from blood samples taken before (pre), directly after (post) and 2 h after (+2 h) the 1‐h treadmill session (*n* = 11). N, normal ambient temperature condition (19.2 ± 0.9°C); H, hot condition (28.7 ± 0.7°C). Statistically significant values are highlighted in bold.

^a^ Significantly different from individual pre‐exercise level.

^b^ Significantly different from respective individual N level (*p* ≤ 0.05).

**FIGURE 3:**
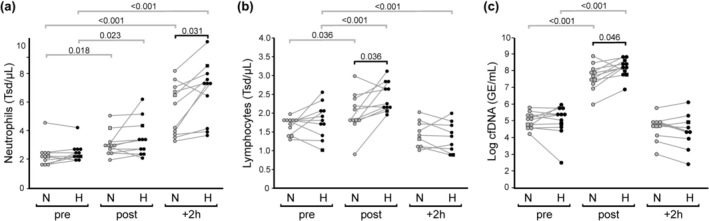
Effect of exercise and ambient temperature on blood levels of circulating neutrophils (a), lymphocytes (b), and cfDNA (c). Illustrated are pairwise comparisons of measured values before (pre), after (post), and 2 h after (+2 h) the 1‐h treadmill session under normal ambient temperature (N, gray symbols) and under moderate heat condition (H, black symbols), respectively (*n* = 11). The female participant is identified by a square symbol. Significant pairwise differences between the two conditions (black lines) as well as in comparison to respective baseline values (gray lines) are indicated by horizontal lines with *p* values.

When treadmill running was performed under moderate environmental heat stress, we observed an exacerbated exercise‐provoked immunological stress response, as indicated by enhanced mobilization of circulating leukocytes and increased release of cfDNA (Figure 3). At 2 h post exercise, numbers of circulating neutrophils were increased by 2.97 Tsd/μL (CI: 1.78–4.17 Tsd/μL, *p* < 0.001) under N condition. Ambient heat stress amplified neutrophil mobilization (H: Δ 2 h vs. pre, 4.00 Tsd/μL, CI: 2.63–5.38 Tsd/μL, *p* < 0.001), resulting in significantly increased neutrophilia (+2 h: N, 5.23 ± 1.95 Tsd/μL; H, 6.47 ± 2.15 Tsd/μL; Δ H vs. N, 1.24 Tsd/μL, CI: 0.37–2.17 Tsd/μL, *p* = 0.031) (Figure 3a). Post‐exercise numbers of circulating lymphocytes became significantly elevated under both conditions (N, Δ post vs. pre, 0.32 Tsd/μL, CI: 0.07–0.58 Tsd/μL, *p* = 0.036; H, Δ post vs. pre, 0.65 Tsd/μL, CI: 0.42–0.90 Tsd/μL, p < 0.001), with a significantly higher mobilization under H condition (post: N, 1.97 ± 0.53 Tsd/μL; H, 2.43 ± 0.39 Tsd/μL; Δ H vs. N, 0.46 Tsd/μL, CI: 0.13–0.80 Tsd/μL, *p* = 0.036) (Figure 3b). Blood levels of cfDNA extensively increased both under N (Δ post vs. pre, 1957 GE/mL [1234 GE/mL, 3000 GE/mL], *p* < 0.001) as well as under H condition (Δ post vs. pre, 3293 GE/mL [2225 GE/mL, 4688 GE/mL], *p* < 0.001). Under moderate heat stress, exercise‐provoked cfDNA response became significantly boosted (post: N, 2046 GE/mL [1336 GE/mL, 3196 GE/mL]; H, 3305 GE/mL [2309 GE/mL, 4895 GE/mL]; *p* = 0.046) (Figure 3c).

The treadmill exercise protocol significantly affected circulating levels of potassium, SCr, LDH, CK, urea, GGT, and AST, but did not influence ALT and sodium (Table 2). Within these parameters, a significant interaction effect of ambient temperature was observed for SCr (*F* (2, 20) = 5.73, *p* = 0.011, $\eta_{p}^{2}$ = 0.371) and potassium (*F* (2, 20) = 6.74, *p* = 0.004, $\eta_{p}^{2}$ = 0.310). Exercise provoked an acute rise in SCr plasma concentrations (N, Δ post vs. pre, 0.16 mg/dL, CI: 0.10–0.21 mg/dL, *p* = 0.003; H, Δ post vs. pre, 0.22 mg/dL, CI: 0.17–0.27 mg/dL, *p* < 0.001) that sustained till 2 h post exercise (N, Δ +2 h vs. pre, 0.13 mg/dL, CI: 0.07–0.19 mg/dL, *p* = 0.003; H, Δ +2 h vs. pre, 0.22 mg/dL, CI: 0.17–0.27 mg/dL, *p* < 0.001). Significant higher SCr levels under H condition were evident immediately after exercise (post: N, 0.9 ± 0.13 mg/dL; H, 1.03 ± 0.12 mg/dL; Δ H vs. N, 0.14 mg/dL, CI: 0.03 to 0.24 mg/dL, *p* = 0.049). Likewise, plasma potassium concentration slightly rose during exercise under H condition from 3.68 ± 0.31 mmol/L to 4.19 ± 0.22 mmol/L (H, Δ post vs. pre, 0.49 mmol/L, CI: 0.21–0.76 mol/L, *p* = 0.028), resulting in significant higher post‐exercise concentrations compared to post‐exercise values under N condition (post: N, 3.99 ± 0.17 mmol/L; H, 4.19 ± 0.22 mmol/L; Δ H vs. N, 0.20 mmol/L, CI: 0.05–0.35 mmol/L, *p* = 0.038).

**FIGURE 4:**
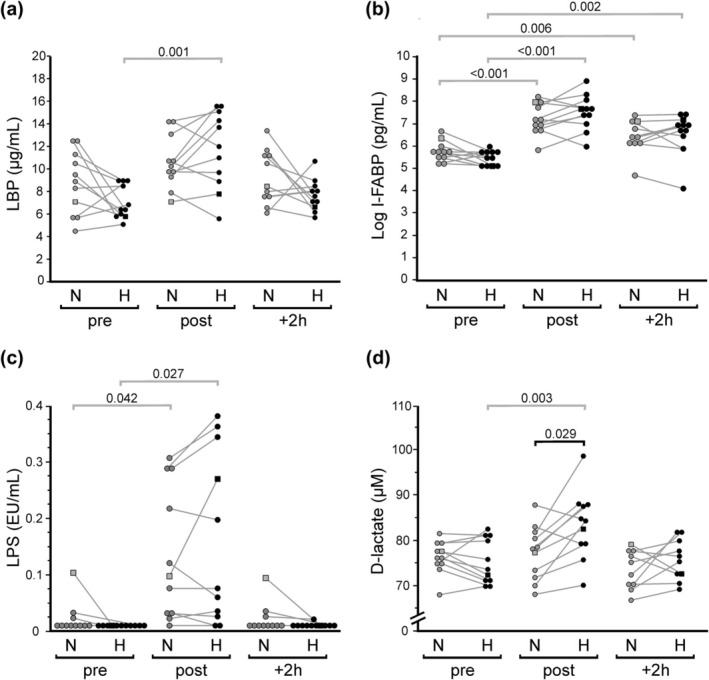
Effect of exercise and ambient temperature on blood levels of LBP (a), I‐FABP (b), endotoxin (c), and D‐lactate (d). Illustrated are pairwise comparisons of measured values before (pre), after (post), and 2 h after (+2 h) the 1‐h treadmill session under normal ambient temperature (N, gray symbols) and under moderate heat condition (H, black symbols), respectively (*n* = 11). The female participant is identified by a square symbol. Significant pairwise differences between the two conditions as well as in comparison to respective baseline values are indicated as horizontal lines with *p* values.

Regarding markers of intestinal impairment, we found LBP, I‐FABP, D‐Lactate, and LPS becoming significantly affected by the exercise intervention protocol (Table 2). In contrast, plasma levels of bacDNA remained unaltered throughout the analyzed time points (*F* (2, 20) = 2.12, *p* = 0.134, $\eta_{p}^{2}$ = 0.105). Post‐exercise plasma levels of LBP became significantly increased under elevated ambient temperature (H, Δ post vs. pre, 5.44 μg/mL, CI: 3.13–7.76 μg/mL, *p* = 0.001), and also trended to increase under normal condition (N, Δ post vs. pre, 1.44 μg/mL, CI: −0.01 to 2.89 μg/mL, *p* = 0.076) (Figure 4a). No significant difference for LBP became apparent by within‐subject comparison of post‐exercise levels from the two conditions (post: N, 10.5 ± 2.35 μg/mL; H, 11.6 ± 3.35 μg/mL; Δ H vs. N, 1.12 μg/mL, CI: −0.81 to 3.04 μg/mL, *p* = 0.224). Exercise also provoked an acute rise in I‐FABP, both at normal temperature (N, Δ post vs. pre, 779 pg/mL [500 pg/mL, 1996 pg/mL], *p* < 0.001) as well as under moderate ambient heat stress (H, Δ post vs. pre, 1761 pg/mL [695 pg/mL, 2827 pg/mL], *p* < 0.001) (Figure 4b). Post‐exercise levels of I‐FABP trended to differ between conditions (post: N, 1102 pg/mL [800 pg/mL, 2492 pg/mL]; H, 1982 pg/mL [1071 pg/mL, 3135 pg/mL]), but within‐subject impact of ambient temperature provided not significant difference (post: Δ H vs. N, 646 pg/mL [−411 pg/mL, 1353 pg/mL], *p* = 0.092). LPS blood concentrations exhibited a rather heterogenous response pattern, with a subset of probands displaying increased levels after exercise (N, Δ post vs. pre, 0.10 EU/mL [0.04 EU/mL, 0.23 EU/mL], *p* = 0.042; H, Δ post vs. pre, 0.14 EU/mL [0.04 EU/mL, 0.23 EU/mL], *p* = 0.027) (Figure 4c). However, within‐subject comparison of post‐exercise values revealed no significant difference between exercising under H and under N condition (post: N, 0.12 EU/mL [0.02 EU/mL, 0.26 EU/mL]; H, 0.15 EU/mL [0.02 EU/mL, 0.32 EU/mL]; *p* = 0.310). In contrast, most probands exhibited significantly higher D‐Lactate blood concentrations after exercising in the heat (post: N, 77.1 ± 5.96 μmol/L; H, 83.2 ± 7.44 μmol/L; Δ H vs. N, 6.08 μmol/L, CI: 1.83–10.3 μmol/L, *p* = 0.029) (Figure 4d). Significant exercise‐provoked release of D‐lactate could be observed under H condition (H, Δ post vs. pre, 8.08 μmol/L, CI: 4.89–11.27 μmol/L, *p* = 0.003) but not under N condition (N, Δ post vs. pre, 1.47 μmol/L, CI: −1.69 to 4.62 μmol/L, *p* = 0.861).

### 3.3 Relationship between core body temperature and markers of inflammation and gastrointestinal distress

In total, 16 athletes (14 males, 2 females; age, 33.9 ± 6.1 years) completed the 1‐h treadmill protocol (main exercise speed: 12.9 ± 1.4 km/h) under moderate heat stress conditions (H: 28.8 ± 0.6°C; relative humidity, 53.7 ± 7.8%). Exercise resulted in a significant elevation of Tc (pre, 37.2 ± 0.3°C; post 39.6 ± 0.5°C; Δ post vs. pre, 2.4°C, CI: 2.0–2.8°C, *p* < 0.001), HR (pre, 71.6 ± 12.4 bpm; post 179.2 ± 7.2 bpm; Δ post vs. pre, 107.6 bpm, CI: 102.7–112.5 bpm, *p* < 0.001), and RPE (pre, 6 [6, 6]; post, 19 [15, 19]; Δ post vs. pre, 12 [9, 13], *p* < 0.001). Total running time at a Tc above 39°C ranged between 0 and 39 min (mean, 15 ± 11 min). Mean estimated water loss by sweating was 1.9 ± 0.45 kg.

**FIGURE 5:**
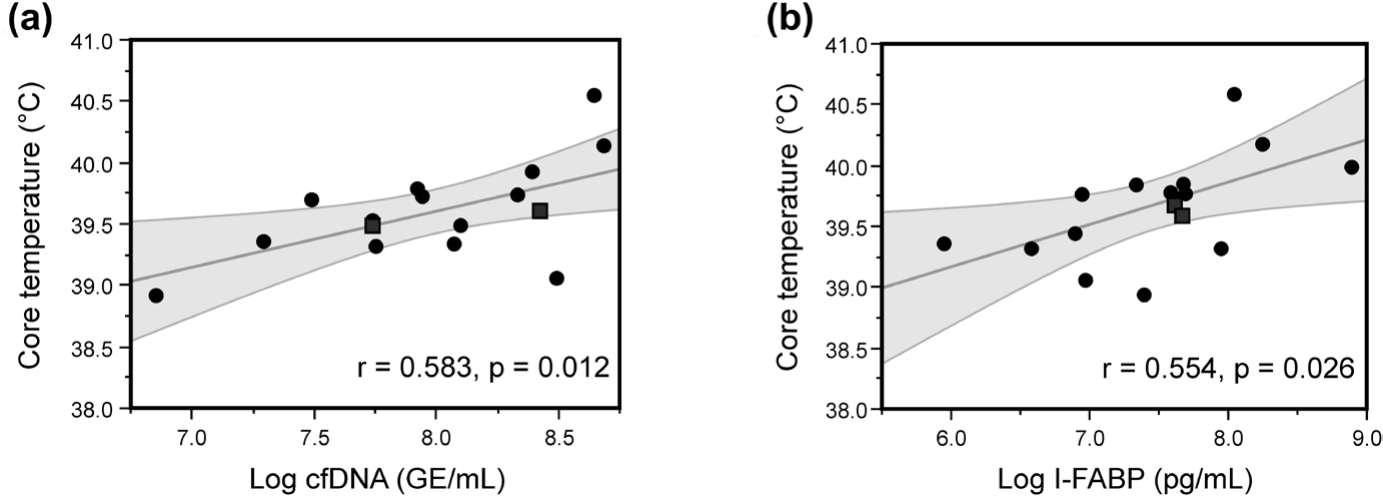
Relationship between Tc, max and post‐exercise values of cfDNA (a) and I‐FABP (b). The female participants are identified by square symbols. The gray shaded areas represent the 95% prediction interval around the linear regression line. Figures include data from 16 participants that completed the 1‐h treadmill session under moderate ambient heat condition (28.8 ± 0.6°C).

Multiple correlation analysis of post‐exercise values (including Tc, max as well as running time at Tc ≥39°C) predicted a significant association between Tc, max and exercise‐provoked release of cfDNA (*r* = 0.583, *p* = 0.012) (Figure 5a). A significant moderate correlation was also apparent for post‐exercise plasma levels of I‐FABP with Tc, max (*r* = 0.554, *p* = 0.026) (Figure 5b) as well as with running time at Tc ≥39°C (*r* = 0.497, *p* = 0.049). There was no apparent correlation between Tc, max and post‐exercise concentrations of LBP (*r* = −0.048, *p* = 0.861) or D‐lactate (*r* = −0.082, *p* = 0.763).

## 4 DISCUSSION

The current study aimed to determine the impact of a vigorous 1‐h endurance exercise session at fixed intensity on humoral markers commonly associated with heat stress and gastrointestinal impair in well‐trained individuals, both at temperate conditions as well as under moderate environmental heat stress. We observed a significant release of several “leaky gut” biomarkers in response to exercise. Exposure to moderate heat during exercise had a slight to moderate accelerating effect on some but not all examined markers. We further observed an impact of ambient condition on plasmatic markers of renal stress, cfDNA release, and on the distribution of immune cell populations. Exercising in moderate heat significantly elevated Tc along the run, with 90% of all participants attaining Tc, max values above the 39°C threshold. The drift in Tc between the two ambient conditions was preceded by a drift in HR responses which was accompanied by higher subjective measures of RPE. Similar Tc and HR curve progressions have previously been reported when monitoring trained individuals during a 2‐h submaximal treadmill run in warm versus temperate ambient conditions (Snipe et al., 2018b).

In accordance with results from a previous study using the same exercise setup, running in moderate heat resulted in an increased exercise‐provoked mobilization and redistribution of circulating leukocytes (Niess et al., 2003), indicating a heightened state of immune surveillance compared with exercising at temperate conditions. Enhanced neutrophilia under hot environmental conditions is a consistently reported hallmark feature in diverse published exercise studies (Mestre‐Alfaro et al., 2012; Mitchell et al., 2002; Severs et al., 1996). However, it is controversial whether the rise in neutrophil counts after exercise is directly connected to increases in Tc, or rather is primarily determined by enhanced adrenergic or cholinergic signaling (Laing et al., 2008). Since hyperthermia is not solely limited to the impact of exercise and environmental conditions but can also arise from endogenous or exogenous pyrogens, neutrophilic responses to elevated Tc are necessarily multifaceted and complex. Several exercise studies report Tc‐dependent changes in markers of neutrophil degranulation, oxidative burst, and antioxidant enzyme defenses, albeit with no consistent picture arising (Laing et al., 2008; Mestre‐Alfaro et al., 2012; Peake et al., 2008). Experimental studies indicate that hyperthermic fever spikes in the course of bacterial infection can trigger a specific emergency response in neutrophils that involves exaggerated release of neutrophil extracellular traps (NETs) (Janko et al., 2023; Keitelman et al., 2019). NETs, web‐like DNA structures of decondensed chromatin and granular proteins, serve as a first‐line defense mechanism against exogenous and endogenous threats. NETs interact with innate and adaptive immunity, provide a physical barrier to prevent systemic spread of pathogens, and are key players in immunothrombosis (Beiter et al., 2015). However, the uncontrolled, excessive, or prolonged release of NETs can exacerbate inflammation, promote tissue damage, and vessel occlusion, thereby contributing to the development and progression of a range of acute and chronic diseases, including several gastrointestinal disorders like inflammatory bowel disease, intestinal ischemia–reperfusion injury, necrotizing enterocolitis, and colorectal cancer (Chen et al., 2022). Moreover, aberrant NET release appears to be the fatal driver of disseminated intravascular coagulation in sepsis (McDonald et al., 2017) as well as in heat stroke (Zhang et al., 2022). Formation of NETs has been demonstrated to become actively triggered in the bloodstream of exercising individuals (Beiter et al., 2014). By use of DNA methylation analysis, it has recently been evidenced that NETs constitute the major source that accounts for the massive exercise‐induced elevation of cfDNA (Fridlich et al., 2023). The same study highlighted Tc as an independent contributor to elevated neutrophil cfDNA in response to exercise. In line with this, here we observed an amplifying impact of higher ambient temperature on exercise‐triggered cfDNA release, as well as a moderately high correlation between post‐exercise levels of cfDNA and individually attained Tc, max.

A positive, albeit moderate, correlation was also observable between Tc, max and plasma levels of I‐FABP at the end of the heat‐stress run. Correlative increases in I‐FABP and Tc are consistently found across those published studies where exercise intensity and/or ambient temperature did suffice to raise Tc values to 39°C and beyond (Henningsen, Mika, et al., 2024; Ogden, Child, et al., 2020; Osborne et al., 2019; Snipe et al., 2018a). FABPs are a group of small intracellular proteins that are abundantly present in different isoforms in the cytosol of most tissues. Several members of the FABP family are known to have very distinct tissue‐specific expression patterns that reflect tissue‐specific functions related to fatty acid disposition and intracellular lipid homeostasis (Storch & Corsico, 2008). I‐FABP exists specifically in mature enterocytes of the small intestinal villi, and thus has been highlighted as a versatile blood biomarker in diverse conditions where mucosal cell membrane integrity may become compromised, particularly in conditions of acute and chronic mesenteric ischemia (Ho et al., 2020). In patients with severe abdominal trauma, I‐FABP plasma concentrations can acutely rise up to 1000‐fold above baseline level (De Haan et al., 2009). Moderate‐to‐high intensity exercise has been reported to acutely increase circulating I‐FABP levels somewhere in the range between 2‐ to 50‐fold (a comprehensive overview of published studies is given by Ogden, Child, et al. (2020)). Nausea, vomiting, cramping, diarrhea, and even gastrointestinal bleeding are common complaints experienced by athletes that participate in endurance competition events (De Oliveira et al., 2014; Papantoniou et al., 2023). Symptoms of gastrointestinal distress amplify with the intensity and duration of exercise, and can be exacerbated in hot ambient conditions (Costa et al., 2017; De Oliveira & Burini, 2009). In the future, the intensity as well as the time of persistence of the exercise‐provoked I‐FABP response might provide valuable prognostic and predictive information for the occurrence, severity, and risk assessment of gastrointestinal complications to aid in improved screening and prevention strategies. However, despite some promising anecdotal reports, the current evidence base is still scarce and more studies are needed to elucidate the meaning and explanatory power of I‐FABP measures regarding exercise‐associated gastrointestinal symptoms (Henningsen, Mika, et al., 2024; Karhu et al., 2017; Kelly et al., 2023; Van Venrooij et al., 2022; Walter et al., 2021; Young et al., 2023). Specifically, we have to understand whether there are critical blood values for I‐FABP that would allow to distinguish between conditions of increased shedding of enterocytes as part of normal gut homeostasis, active release of I‐FABP (as has been reported for other FABP isoforms (Bronsky et al., 2006; Kralisch et al., 2015; Mita et al., 2015)), and significant structural damage to the intestinal mucosa.

At present, it is also not clear whether increased I‐FABP levels in response to exercise are reliably predictive of increased intestinal permeability. Only a few studies directly addressed this issue by use of dual sugar absorption tests. Although direct comparison of findings is limited by disparities in research questions and study designs, these studies generally indicate rather weak correlations between gut permeability and I‐FABP responses (March et al., 2017; Ogden, Fallowfield, et al., 2020; Van Wijck et al., 2011, 2012). Intestinal permeability is tightly regulated by junctional complexes that seal together adjacent epithelial cells, provide cytoskeletal anchorage, and regulate the passage of ions, small water‐soluble compounds, and water itself through the paracellular spaces (Horowitz et al., 2023). They form a highly dynamic entity that can be affected by various endogenous and exogenous factors. Hyperthermia, whether exertional or environmental, in conjunction with exercise elicits a complex regime of gastrointestinal stress where multiple stressors (i.e., thermal stress, hormonal stress, metabolic stress, oxidative stress, hypoxia, and mechanical stress) combine simultaneously at varying degrees to affect intestinal integrity and tight junction permeability. By use of dual sugar absorption tests, enhanced passage of intraluminal molecules has been demonstrated to become triggered by intensive endurance exercise and to become amplified when exercise was performed under environmental heat stress (Fung et al., 2021; Ogden, Child, et al., 2020; Pires et al., 2017). Increased paracellular permeability via the so‐called leak pathway is a normal physiological response that supports absorption of nutrients when carrier‐mediated transcellular transport is saturated, but it is also a contributor to gastrointestinal dysfunction and symptoms in various pathological conditions (Grover et al., 2024; Horowitz et al., 2023). Passage through the tight junction leak pathway describes a route in which small organic molecules with hydrated diameters up to 100 Å can move across the mucosal barrier, albeit in limited capacity (Shen et al., 2011). In contrast, the high‐capacity pore pathway allows only the passage of small, charged ions (upper limit of 6 to 8 Å) and water molecules. Both routes can be independently regulated, but both routes lack the structural specificity of transcellular transport. Thus, paracellular absorption via the leak pathway is not stereospecific and can also accommodate small molecules for which there is no apical transporter, like the bacterial fermentation product D‐Lactate (hydrated molecular size: <50 Å) (Horowitz et al., 2023). Blood D‐lactate has been found elevated in patients with metabolic disorders and gastrointestinal conditions such as short bowel syndrome, acute mesenteric ischemia, or gastrointestinal dysfunction (Cai et al., 2019; Talasniemi et al., 2008; Teng et al., 2021). The increased D‐lactate levels we measured after running under moderate heat conditions point towards instigation of the leak pathway and increased intestinal permeability compared to running at temperate conditions. However, we have to consider that the reliability of absolute D‐lactate values as a quantitative marker of gut permeability may be compromised by the individual composition of the gut microbiota and the relative abundance of D‐lactate‐producing bacteria therein (Snelson et al., 2024).

The premise underlying the “leaky gut” hypothesis is that increased mucosal permeability in response to intense exercise and/or ambient heat stress not only allows passage of small microbial metabolic products but also provides an entrance gate for intestinal bacteria, bacterial compounds, and bacterial toxins into the lamina propria as well as into the systemic circulation, provoking local and systemic inflammation that increases the risk of acute and chronic health hazards (Costa et al., 2017; Dmytriv et al., 2024; Ogden, Child, et al., 2020; Ribeiro et al., 2021). A potential biomarker that has been proposed to indicate mucosal barrier breach and bacterial translocation is the secretory acute‐phase protein LBP (Perez‐ Diaz‐Del‐Campo et al., 2023). LBP is named after its ability to bind to LPS of Gram‐negative bacteria, but it also can recognize other bacterial compounds, such as glycolipids and lipopeptides (Schroder & Schumann, 2005). It is mainly produced in the liver and strongly modulates the response to endotoxins (Ryu et al., 2017). LBP plasma levels increase dramatically after bacterial challenges (Zweigner et al., 2001) but also have been shown to acutely respond, albeit to a much lesser extent, in conditions of controlled hyperthermia (Lee et al., 2024; Mckenna et al., 2024) and hypoxia (McKenna et al., 2023). LBP has further been implemented as a marker for metabolic endotoxemia in inflammatory metabolic conditions such as atherosclerosis, obesity, and type 2 diabetes (Liu et al., 2014; Moreno‐Navarrete et al., 2012; Sakura et al., 2017). In line with our findings, moderately increased circulating LBP levels in response to exercise have been reported after completion of 2 h of moderate endurance running (Young et al., 2024) or high‐intensity interval exercise in the heat (Mckenna et al., 2022). However, we have to emphasize that increase in LBP does not necessarily equal with increase in endotoxin. LBP also functions as an antioxidant and mediator of lipid metabolism that participates in redox signaling pathways to counteract liver oxidative stress in infectious as well as in noninfectious conditions (Milbank et al., 2023; Song et al., 2021; Zhang et al., 2024). The unique function of the liver to supply glucose for the working muscle renders this organ especially susceptible for exercise‐induced oxidative stress that is aggravated with rising Tc (Garcin et al., 2008; Hoene & Weigert, 2010). Elevation of serum transaminase AST but not ALT following exercise, as observed in our study, may indicate an acute imbalance in liver redox state, albeit we also have to consider skeletal muscle as a primary source of AST release. Necessarily, any gut luminal content, including endotoxins or microorganisms, that should cross the gut barrier and enter the gut capillary network will be delivered to the liver via the portal vein (Pabst et al., 2023). Here, physiological amounts of potentially harmful or immunogenic agents will either become degraded, removed, or detoxified before reaching the peripheral circulation. Whether the liver's capacity to systemically clear endotoxin becomes temporarily affected by strenuous exercise, to our knowledge, has not been investigated so far.

Several studies have attempted to measure exercise‐provoked changes in peripheral blood endotoxin concentrations over a range of exercise durations and intensities under varying ambient conditions (Ashton et al., 2003; Barberio et al., 2015; Gill, Hankey, et al., 2015; Gill, Teixeira, et al., 2015; Lim et al., 2009; Nieman et al., 2006; Snipe et al., 2018b; Yeh et al., 2013; Zuhl et al., 2015). Most, but not all, studies describe a slight to moderate increase in circulating plasma LPS levels post exercise, and it appears that exercising at higher intensity, longer duration, or under heat exposure may amplify this effect. However, in congruence with our observations, all these studies describe a large interindividual range of endotoxin responses that makes it difficult to draw any firm conclusions about causation. It has to be considered that host‐specific differences in origin, structure, solubility, physical state, and bioactivity of bloodborne LPS can profoundly influence the outcome of endotoxin assays and may preclude accurate comparisons of endotoxin levels among individuals and studies (Munford, 2016). An additional potential marker of the circulating load of bacterial fragments could be bacDNA that, in contrast to endotoxin assays, would allow for detection of both Gram‐positive and Gram‐negative bacteria. Increased levels of bacDNA have been reported in plasma of various cohorts of patients with infectious and noninfectious diseases, including sepsis, human immunodeficiency virus (HIV)–infection, inflammatory bowel disease, metabolic and cardiovascular diseases, cancer, and pneumonia (Pietrzak et al., 2023). Reported correlations between plasma endotoxin and bacDNA, however, are commonly weak and inconsistent. So far, few studies have measured the plasma concentration of bacDNA in response to exercise. It appears that exercise trials of moderate duration or submaximal intensity, as in our study, have no significant effect on plasma levels of bacDNA, irrespective of ambient temperature (March et al., 2019; Ogden, Fallowfield, et al., 2020). In contrast, circulating bacDNA concentrations have been reported to become affected when measured after more prolonged and more demanding exercise interventions and under exertional heat stress (Gaskell et al., 2023; Henningsen, Henry, et al., 2024; Young et al., 2023). Whether these observations truly relate to increased bacterial translocation from the gastrointestinal tract or other microbiome niches within the body requires further investigation.

Two important yet unsolved questions arise when discussing the potential of exercise‐provoked endotoxemia. Firstly, by which route should bacteria or endotoxins translocate from the gut lumen into the systemic circulation? Second, is the endotoxin that can be detected in the plasma of exercising individuals truly bioactive in vivo? At present state, no direct evidence exists supporting the transport of large and lipophilic compounds like endotoxins, let alone whole bacteria, through the paracellular leak pathway (Hollander & Kaunitz, 2020; Munford, 2016; Quigley, 2016). Therefore, current models propose substantial epithelial cell disruption and a breach in epithelial cell layer integrity owing to the combined impact of thermal, oxidative, mechanical, and endoplasmic reticulum stress, resulting in unrestricted flux of LPS molecules, macromolecular complexes, and even intact bacteria (Henningsen, Martinez, & Costa, 2024; Lian et al., 2020; Ogden, Child, et al., 2020). In fact, substantial loss of intestinal epithelium has been demonstrated in experimental animal models when Tc rises above 42°C (Sun et al., 2024). Likewise, mucosal erosions and gastrointestinal bleeding as observed by endoscopy in competitive long‐distance runners might be indicative for enhanced risk of cell damage and loss of epithelial integrity following prolonged strenuous endurance exercise (Papantoniou et al., 2023). However, it is not plausible to assume that submaximal exercise under moderate heat stress, as applied in our study, should induce substantial disruption of tight junctions, mucosal cell death, and gaps in the epithelial layer of asymptomatic healthy athletes. In recent years, several transcellular routes have been deciphered that would allow passage of large immunogenic molecules, particulates, or microorganisms across intact intestinal epithelial cells (Guerville & Boudry, 2016). It has been demonstrated that in conditions of metabolic endotoxemia or following consumption of a high‐fat diet, endotoxins can cross the intestinal barrier via lipid raft‐mediated endocytosis in the course of fatty acid absorption by enterocytes (Akiba et al., 2020; Ghoshal et al., 2009). Furthermore, goblet cells and M cells are specifically equipped to take up soluble antigens, particulate antigens, and microorganisms from the gut lumen and transport them via transcytosis across the epithelium to the antigen‐presenting cells underneath (Dillon & Lo, 2019; Zhang et al., 2023). It is important to note that these transcellular transport pathways are tightly connected to diverse intrinsic and downstream detoxification mechanisms that diminish or attenuate the immunogenicity of gut‐derived endotoxins and/or promote rapid clearance of LPS and other luminal‐derived bacterial products (Guerville & Boudry, 2016). One might even speculate that acute triggering of these transport pathways may help to maintain gut immune homeostasis by balancing immune surveillance and tolerance as a supportive explanatory model for the diverse health‐promoting effects of moderate endurance exercise in disease prevention and therapy. Necessarily, this system could be overloaded when the body's inherent cooling system becomes overwhelmed during high‐intensity or long‐duration exercise in the heat. Excessive or uncontrolled translocation of endotoxins, overwhelmed clearance, and impaired detoxification mechanisms in conjunction with an unfavorable gut bacteria composition and LPS types could then provoke local adverse inflammatory reactions that may eventually lead to gut barrier deterioration, dysregulated systemic inflammation, excessive NET formation, spread of intravascular coagulation, and organ damage, with fatal outcomes like exertional heat illnesses and heat stroke (Garcia et al., 2022). The exact sequel of events and the critical breaking points in this scenario have yet to be deciphered.

### 4.1 Limitations

The present study has certain limitations that should be acknowledged. Of the 18 participants included in this study, only data from 11 subjects could be considered for paired analysis, mainly due to a limitation of the air‐conditioning system to maintain ambient temperature below the 21°C limit in the N condition trial. Our air‐conditioning equipment did not allow us to maintain relative humidity within a constant range. Moreover, due to logistical challenges, the overall timeframe of the study included exercise tests from autumn to spring. Individual responses might therefore be biased due to seasonal variations in training loads and heat acclimatization. Thus, our study setup is not sufficiently standardized and is underpowered to allow for comparisons between subjects or to account for the impact of age, gender, cardiovascular fitness, or body composition on individual responses to heat and exercise. Moreover, the applied exercise protocol did not suffice to provoke moderate or even severe gastrointestinal symptoms. Therefore, we have no quantitative clinical outcome measures to provide qualitative insights into the discriminatory or predictive power of measured blood variables.

## 5 CONCLUSIONS

In summary, our study shows that a broad range of so‐called “leaky gut” biomarkers are released into the blood stream during an acute bout of strenuous exercise, and this response becomes increased when the endurance exercise is performed under elevated ambient temperature. To avoid confusion caused by the pervasive indiscriminate use of the terms “leaky gut”, “intestinal permeability”, and “intestinal barrier disruption”, it appears crucial to clearly define the role of each marker in the complex chain of processes that maintain gut and whole‐body homeostasis when challenged by thermal, oxidative, metabolic, mechanical, and inflammatory stress. We further argue that also transcellular transport pathways should be considered when addressing the question of exercise‐induced endotoxemia.

## 6 FUTURE DIRECTIONS

Human physiology has specifically been shaped by evolution within the past 2 million years to withstand protracted locomotion in high‐temperature environments (Lieberman, 2015). To get a better understanding of what makes modern humans probably more vulnerable to exercising under heat stress than our hunter‐gatherer ancestors, we have to look beyond our inherited genomic equipment and integrate the thousands of genes that are additionally provided by our commensal microbiota. The balanced ecosystem of host and microbiome has experienced several dramatic challenges during the last 15,000 years when Homo sapiens created the Anthropocene by starting neolithic, industrial, and hygienic revolutions with profound impacts on overall dietary, locomotion, and social stress patterns. The role of the microbiome in health and diseases has been highlighted by numerous studies, and alterations in the composition of the gut microbiota are tightly connected to the global epidemic burden of noncommunicable diseases, including obesity, diabetes, hypertension, cardiovascular diseases, cancer, allergies, and neuropsychiatric disorders (Hou et al., 2022). There is also increasing evidence that the gut microbiome has a profound impact on athletic performance, and, vice versa, that acute and regular exercise can profoundly alter the composition of the gut microbiota (O'brien et al., 2022). Recent animal studies reveal a very intricate and reciprocal interplay between gut microbiome and short‐ as well as long‐term adaptations in response to the combined action of exercise and heat stress (Dohnalova et al., 2022; Kim et al., 2023; Valentino et al., 2021; Wen et al., 2021). Research in this area will open novel avenues for athletes as well as for the general population to better cope with a continuously warming environment where extreme heatwaves should be expected to become more the rule than the exception. However, at present we are still far from a clear understanding of what defines a “healthy” microbiome, let alone a “performance‐enhancing” or a “heat‐tolerable” microbiome (Fan & Pedersen, 2021).

## FUNDING INFORMATION

This project was supported by a grant from the German Federal Institute of Sports Sciences (Bundesinstitut für Sportwissenschaft, BISP), Project Number: ZMI4‐070112/21‐22. We further acknowledge support from the Open Access Publishing Fund of the University of Tübingen.

## CONFLICT OF INTEREST STATEMENT

The authors have no conflicts of interest to declare.

## ETHICS STATEMENT

This study was approved by the local ethics committee of the University of Tübingen (approval number: 008/2022BO2); written informed consent was obtained from all participants, and all procedures and protocols conformed to the standards of use of human participants in research as outlined in the Sixth Declaration of Helsinki.

## Data Availability

The raw data supporting the conclusion of this article will be made available by the authors, without undue reservation.
